# Population structure and geographical segregation of *Cryptosporidium parvum* IId subtypes in cattle in China

**DOI:** 10.1186/s13071-020-04303-y

**Published:** 2020-08-18

**Authors:** Zhenjie Zhang, Suhui Hu, Wentao Zhao, Yaqiong Guo, Na Li, Zezhong Zheng, Longxian Zhang, Martin Kváč, Lihua Xiao, Yaoyu Feng

**Affiliations:** 1 Center for Emerging and Zoonotic Diseases, College of Veterinary Medicine, South China Agricultural University, Guangzhou, Guangdong 510642 China; 2 Guangdong Laboratory for Lingnan Modern Agriculture, Guangzhou, 510642 China; 3 College of Animal Science and Veterinary Medicine, Henan Agricultural University, Zhengzhou, 450002 China; 4 Institute of Parasitology, Biology Centre of the Academy of Sciences of the Czech Republic, České Budějovice, 37005 Czech Republic

**Keywords:** *Cryptosporidium parvum*, Epidemic, Geographical segregation, Cattle, China

## Abstract

**Background:**

*Cryptosporidium parvum* is a zoonotic pathogen worldwide. Extensive genetic diversity and complex population structures exist in *C. parvum* in different geographical regions and hosts. Unlike the IIa subtype family, which is responsible for most zoonotic *C. parvum* infections in industrialized countries, IId is identified as the dominant subtype family in farm animals, rodents and humans in China. Thus far, the population genetic characteristics of IId subtypes in calves in China are not clear.

**Methods:**

In the present study, 46 *C. parvum* isolates from dairy and beef cattle in six provinces and regions in China were characterized using sequence analysis of eight genetic loci, including *msc6-7*, *rpgr*, *msc6-5*, *dz-hrgp*, *chom3t*, *hsp70*, *mucin1* and *gp60*. They belonged to three IId subtypes in the *gp60* gene, including IIdA20G1 (*n* = 17), IIdA19G1 (*n* = 24) and IIdA15G1 (*n* = 5). The data generated were analyzed for population genetic structures of *C. parvum* using DnaSP and LIAN and subpopulation structures using STRUCTURE, RAxML, Arlequin, GENALEX and Network.

**Results:**

Seventeen multilocus genotypes were identified. The results of linkage disequilibrium analysis indicated the presence of an epidemic genetic structure in the *C. parvum* IId population. When isolates of various geographical areas were treated as individual subpopulations, maximum likelihood inference of phylogeny, pairwise genetic distance analysis, substructure analysis, principal components analysis and network analysis all provided evidence for geographical segregation of subpopulations in Heilongjiang, Hebei and Xinjiang. In contrast, isolates from Guangdong, Shanghai and Jiangsu were genetically similar to each other.

**Conclusions:**

Data from the multilocus analysis have revealed a much higher genetic diversity of *C. parvum* than *gp60* sequence analysis. Despite an epidemic population structure, there is an apparent geographical segregation in *C. parvum* subpopulations within China. 
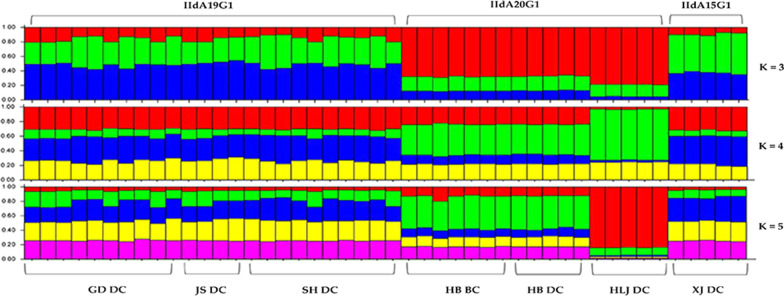

## Background

*Cryptosporidium* spp. are apicomplexan pathogens that can cause debilitating gastrointestinal illness in animals and humans with the main clinical symptom as diarrhea [1]. There is extensive genetic variation within the genus *Cryptosporidium*. Among the nearly 40 *Cryptosporidium* species identified, *C. parvum* is the most important species causing zoonotic cryptosporidiosis [2]. It has a wide host range, with over 20 subtype families based on sequence analysis of the 60 kDa glycoprotein (*gp60*) locus [3]. Among the most common subtype families, IIa and IId are zoonotic, while IIc and IIe are anthroponotic [2, 4].

Cattle are among the most common hosts of *C. parvum*, with pre-weaned calves being considered the most important reservoir for zoonotic *C. parvum* infection [5]. Differences in virulence and transmission dynamics of *C. parvum* have been observed among geographical regions [6]. Subtyping of *C. parvum* in bovine studies identified an exclusive occurrence of IId subtypes in calves in China, mostly IIdA15G1 and IIdA19G1 [7]. Moreover, these IId subtypes have caused outbreaks of cryptosporidiosis in calves in several areas in China, leading to the occurrence of significant mortality [8, 9]. In contrast, pre-weaned calves in industrialized countries are mostly infected with *C. parvum* IIa subtypes, especially IIaA15G2R1 [6, 7].

Population genetic studies based on highly polymorphic loci can shed light on the true genetic diversity of *C. parvum* in disease endemic areas and compensate for the relatively low resolution of the single *gp60* locus because of the likely occurrence of genetic recombination among loci and the existence of genetic determinants of other phenotypic traits [3, 10]. Multilocus typing tools based on genetic loci with simple tandem repeats have been used in studies of the population genetic characteristics of *C. parvum*, leading to the discovery of high genetic diversity, significant geographical segregation and complex population structure [11, 12]. Thus far, a range of genetic structures of *C. parvum* have been identified, including panmictic (unrestricted gene flow and linkage equilibrium among loci), clonal (largely restricted gene flow and linkage disequilibrium among loci), and epidemic (underlying panmictic structure masked by an abundance of genetically identical clones) [2].

Most previous studies of the population genetics of *C. parvum* had focused on the IIa subtype family. A mostly panmictic population structure for *C. parvum* IIa subtype family has been found in humans and calves in many industrialized nations [12–21]. This could be related to the transmission intensity and reproductive characteristics of the IIa subtype family. Indeed, IIa subtypes, especially the hyper-transmissible IIaA15G2R1, are the dominant ones in cattle and humans in these countries [2]. In addition, one study of IIaA15G2R1 has also shown an epidemic population structure and common occurrence of genetic recombination within the subtype [16]. Several analyses of the IId subtype family have demonstrated potential differences in population structure between IId and IIa subtype families. For example, IIa subtypes in cattle in Spain has a panmictic structure while IId subtypes in sheep has a clonal structure [20, 22]. This was supported by a population genetic study of the *C. parvum* IId subtype family in China, Egypt and Sweden, which mostly has a clonal population structure.

The aim of this study was to explore the population genetic characteristics of IId subtypes of *C. parvum* in cattle in China using multilocus sequence typing (MLST) of isolates.

## Methods

### Sample sources

**Fig. 1:**
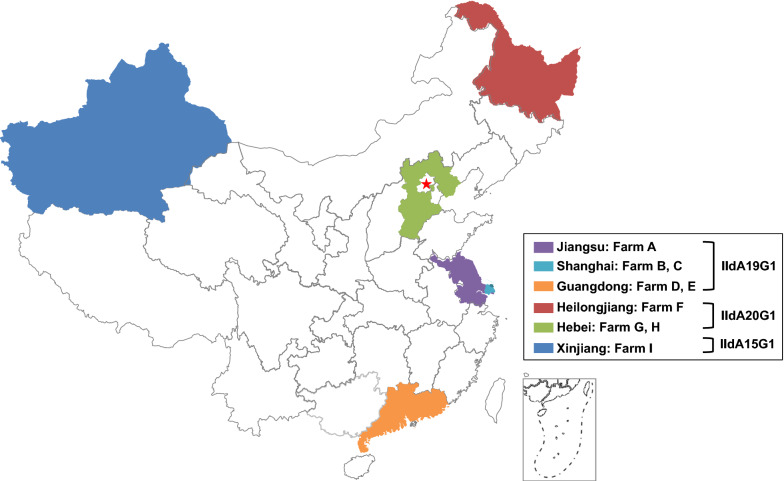
Geographical locations of *Cryptosporidium parvum* samples from dairy and beef cattle in this study in China

Forty-six isolates of *C. parvum* IId subtypes including IIdA20G1 (*n* = 17), IIdA19G1 (*n* = 24), IIdA15G1 (*n* = 5) from beef and dairy cattle in Xinjiang, Heilongjiang, Hebei, Shanghai, Jiangsu and Guangzhou, China, were selected for the population genetics analysis. They were from previous and ongoing studies of molecular epidemiology of cryptosporidiosis in cattle [8, 23, 24]. The geographical distribution of isolates and their *gp60* subtype designations are shown in Table 1 and Fig. 1. The six provinces and autonomous regions are representative ones in China, including the south (Guangdong), east (Shanghai and Jiangsu), center (Hebei), northeast (Heilongjiang) and northwest (Xinjiang). These areas have some of the largest dairy farms in China. The three *C. parvum* subtypes examined in the study are the most common subtypes in China, responsible for over 90% *C. parvum* infections in cattle. They were diagnosed by DNA sequence analysis of the *gp60* gene [23].

**Table 1:** Subtypes and regional origins of *Cryptosporidium parvum* isolates used in the present study

Location	Farm	Host	Subtypes	No. of isolates	Reference
Guangdong	D	Dairy cattle	IIdA19G1	5	[23]
	E	Dairy cattle	IIdA19G1	5	Present study
Hebei	G	Dairy cattle	IIdA20G1	5	Present study
	H	Beef cattle	IIdA20G1	7	Present study
Heilongjiang	F	Dairy cattle	IIdA20G1	5	Present study
Jiangsu	A	Dairy cattle	IIdA19G1	4	[8]
Shanghai	B	Dairy cattle	IIdA19G1	5	[24]
	C	Dairy cattle	IIdA19G1	5	[24]
Xinjiang	I	Dairy cattle	IIdA15G1	5	Present study
Total		Dairy cattle, Beef cattle	IIdA19G1, IIdA20G1, IIdA15G1	46

### PCR and sequence analyses

Eight polymorphic loci including *gp60* with simple tandem repeats were used in the characterization of *C. parvum* isolates in the present study. In addition to *gp60*, they included *msc6-7* (serine repeat antigen), *rpgr* (retinitis pigmentosa GTPase regulator), *msc6-5* (hypothetical trans-membrane protein), *dz-hrgp* (hydroxyproline-rich glycoprotein), *chom3t* (T-rich gene fragment), *hsp70* (70 kDa heat shock protein), *mucin1* (mucin-like protein). Nested PCR was used in the analysis of these genetic loci as previously described [25]. Each isolate was analyzed twice by PCR at each genetic locus. Reagent-grade water was used as a negative control, whereas DNA of *C. parvum* IOWA isolate (IIaA15G2R1 subtype) was used as a positive control. Positive PCR products were sequenced on an ABI 3730 Genetic Analyzer (Applied Biosystems, CA, USA). The sequences generated were assembled using ChromasPro v.2.1.8 (http://technelysium.com.au/ChromasPro.html) and aligned with reference sequences from each locus using the program Clustal X v.2.1 (http://www.clustal.org/).

### Population genetic analyses

The sequences from the eight loci were tandemly concatenated for each isolate. The multilocus genotypes (MLGs) with the same sequences were analyzed for gene diversity (*Hd*), linkage disequilibrium (*LD*) and recombination events (*Rms*) using software DnaSP version 6.12.03 (http://www.ub.edu/dnasp/) with consideration of both sequence length polymorphism and nucleotide substitutions [26]. The genetic structure of *C. parvum* IId subtypes was assessed by measuring the association of standard correlation index ($I_{A}^{S}$) and the relationship between *V*_*D*_ and *L* using the online software LInkage ANalysis, v.3.7 (http://guanine.evolbio.mpg.de/cgi-bin/lian/lian.cgi.pl/query) [27].

### Substructure analyses

Maximum likelihood analysis implemented in the software RAxML v.8.0.0 (http://epa.h-its.org/raxml/submit_single_gene) was used in clustering nucleotide sequences of all isolates using the General Time Reversible (GTR) model [28]. Subpopulations within the 46 isolates of the *C. parvum* IId subtype family were identified using STRUCTURE v.2.3.4 (http://web.stanford.edu/group/pritchardlab/structure.html) [29]. Several analyses of allelic data were performed by using K (likely populations) ranging from 2 to 10 and 50,000 iterations after a ‛burn-in’ of 50,000 iterations. Output at K = 3–5 provided the best fit to MLST data and was used in further analyses. Pairwise genetic distance (*F*_st_) was calculated using Arlequin v.3.5 (http://cmpg.unibe.ch/software/arlequin3/) in the evaluation of the genetic differentiation between MLGs of *C. parvum*. Principal coordinates analysis (PCoA) *via* covariance matrix with data standardization was performed on the generated matrices with the software GENALEX v.6.501 (http://biology-assets.anu.edu.au/GenAlEx) [30]. A median-joining phylogeny was generated using Network software v.5.0 (www.fluxus-engineering.com/sharenet.htm) to estimate the genetic segregation and evolutionary trend of *C. parvum* [31].

## Results

### MLST subtypes and sequence polymorphism

Forty-one of the 46 isolates were successfully amplified at all eight loci. Among them, *dz-hrgp*, *rpgr* and *mucin1* had relatively higher sequence polymorphism, with 5, 4 and 4 subtypes being identified, respectively. In contrast, the 44 isolates generated the same sequence at the normally polymorphic *chom3t* locus (Additional file 1: Table S1). Altogether, 17 MLGs were obtained from these isolates of *C. parvum*. Among them, the IIdA19G1 isolates from Guangdong, Jiangsu and Shanghai consisted of 12 MLGs. In addition, the IIdA20G1 isolates from Hebei and Heilongjiang had two geographically segregated MLGs. The IIdA15G1 isolates from Xinjiang had 3 different MLGs (Additional file 1: Table S1).

Sequence data of all eight loci were concatenated to make a multilocus contig of 4740 bp in length. There was a high genetic diversity (*Hd* = 0.89) within *C. parvum* IId population in China (Table 2). Among the IIdA19G1 isolates, the genetic diversity of isolates from Shanghai (*Hd* = 0.94) was greater than isolates from Guangdong (*Hd* = 0.78) or Jiangsu (*Hd* = 0.67) (Table 2). This could be attributed to the difference in the number of farms examined in different regions. In contrast, IIdA20G1 isolates had relatively low genetic diversity (*Hd* = 0.48). Among them, isolates from Hebei and Heilongjiang showed high genetic homogeneity (*Hd* = 0.00) within each population. In contrast, IIdA15G1 isolates from Xinjiang were highly heterogeneous (*Hd* = 1.00) (Table 2).

**Table 2:** Genetic diversity within *Cryptosporidium parvum* populations based on analysis of the concatenated sequences from eight genetic loci

Population	*n*	*H*	*Hd*	LD (|D’|)	*Rms*
Location					
Guangdong	10	6	0.78	Y = 1.0000 + 0.0000X	0
Hebei	10	1	0.00	–	–
Heilongjiang	5	1	0.00	–	–
Jiangsu	4	2	0.67	–	0
Shanghai	9	7	0.94	Y = 0.9295 + 0.0350X	1
Xinjiang	3	3	1.00	Y = 1.0000 + 0.0000X	0
*gp60* subtypes					
IIdA19G1	23	12	0.85	Y = 0.8811 + 0.0177X	1
IIdA20G1	15	2	0.48	–	0
IIdA15G1	3	3	1.00	Y = 1.0000 + 0.0000X	0
Total	41	17	0.89	Y = 0.9994 −0.0041X	1

*Abbreviations*: *n* number of multilocus genotypes, *H* number of haplotypes (types based on SNPs alone), *Hd* gene diversity; LD (*ǀ*D’*ǀ*), linkage disequilibrium between sites, where X is the nucleotide distance (measured in kilobases); *Rms* minimum number of recombination events

### Population structure of IId subtypes of *C. parvum*

In the analysis of the genetic structure of IId subtypes with *V*_*D*_ and *L* measurements, an epidemic genetic structure was obtained in the overall population (*I*^*S*^_*A*_ = −0.0421, *P*_*MC*_ = 0.889 and *V*_*D*_: 1.1307 < *L*: 2.3307) (Table 3). In further analyses, most of the subpopulations by region or *gp60* subtype also had the epidemic genetic structure, except for the subpopulations of Heilongjiang, Hebei and Xinjiang which could not be determined due to the small sample size (Table 3).

**Table 3:** Results of linkage disequilibrium analysis of allelic profile data from *Cryptosporidium parvum* at eight genetic loci

Populations	*n*	*I*^*S*^_*A*_	*P*_*MC*_	*V*_*D*_	*L*	*V*_*D*_ >* L*	LD or LE
Guangdong	10	−0.0137	0.537	0.7091	1.3455	No	LE
Hebei	12	−0.0110	1	0.2564	0.5641	No	LE
Heilongjiang	5	–	1	–	–	–	–
Jiangsu	4	0.0286	1	0.2667	0.2667	No	LE
Shanghai	10	0.0816	0.067	1.9727	2.0182	No	LE
Xinjiang	5	−0.0128	0.472	1.2111	3.2111	No	LE
IIdA19G1	24	0.0272	0.175	1.2794	1.483	No	LE
IIdA20G1	17	0.0867	< 0.001	0.9638	0.8008	Yes	LD
IIdA15G1	5	−0.0128	0.472	1.2111	3.2111	No	LE
Total	46	0.0672	< 0.001	2.202	1.8848	Yes	LD
Guangdong^a^	6	−0.051	0.898	0.6	1.7429	No	LE
Hebei^a^	2	–	–	–	–	–	–
Heilongjiang^a^	1	–	–	–	–	–	–
Jiangsu^a^	2	–	< 0.001	–	–	–	–
Shanghai^a^	7	−0.0158	0.589	0.9476	2.2476	No	LE
Xinjiang^a^	3	0.2321	0.029	2.3333	2.3333	No	LE
IIdA19G1^a^	12	−0.0695	0.986	0.5979	1.8287	No	LE
IIdA20G1^a^	2	–	< 0.001	–	–	–	–
IIdA15G1^a^	3	0.2321	0.029	2.3333	2.3333	No	LE
Total^a^	17	−0.0421	0.889	1.1307	2.3307	No	LE

^a^Considering isolates with the same MLG as one individual

*Abbreviations*: *n* number of isolates; $I_{A}^{S}$, standardized index of association calculated using the program LIAN 3.5; *P*_*MC*_, significance for obtaining this value in 1000 simulations using the Monte Carlo method; *V*_*D*_, variance of pairwise difference; *L*, 95% critical value for *V*_*D*_; *V*_*D*_ > *L*, presence of linkage disequilibrium

### Subpopulations of IId subtypes of *C. parvum*

**Fig. 2:**
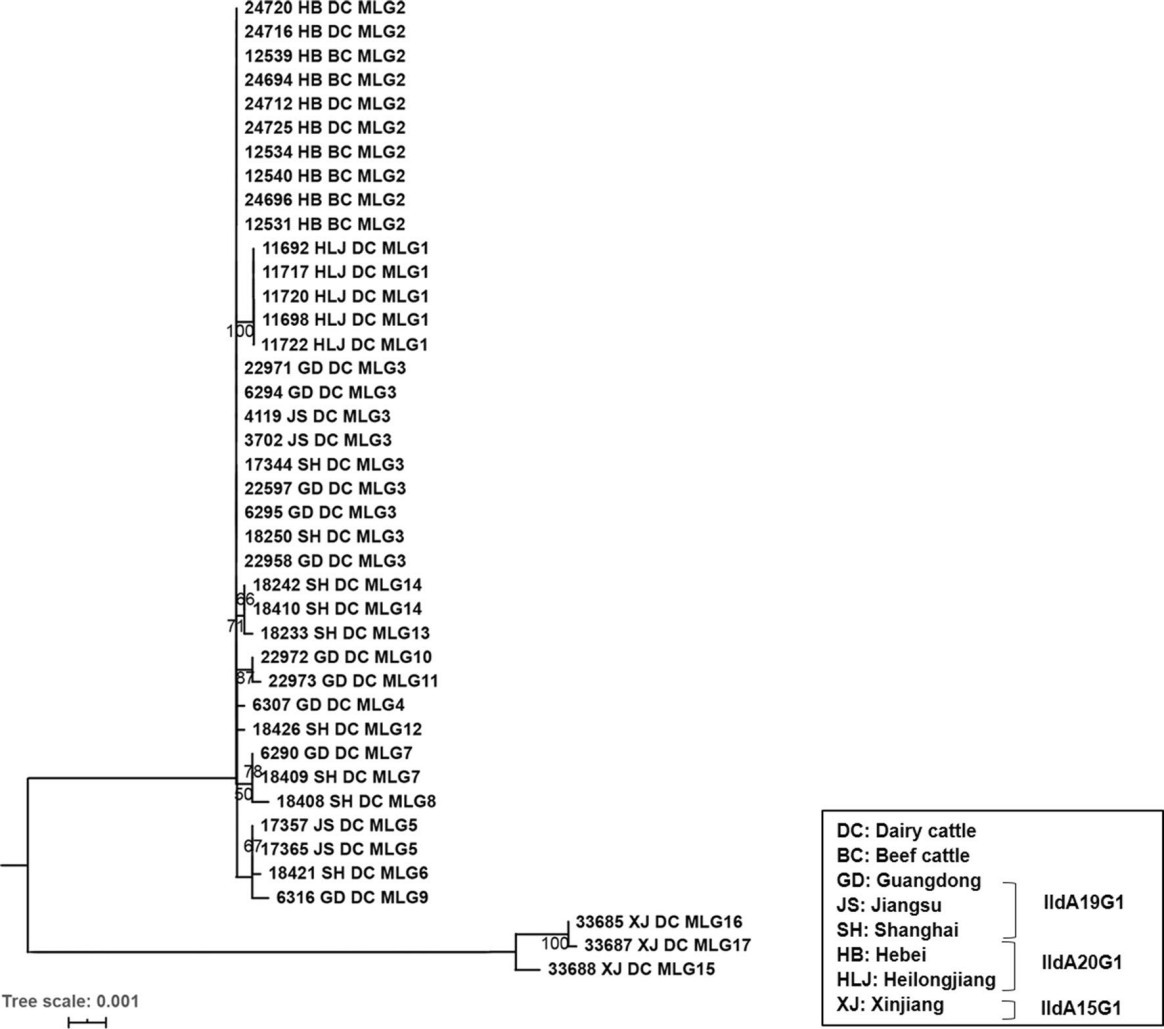
Phylogenetic relationships among 41 *Cryptosporidium parvum* isolates. The phylogeny was inferred through maximum likelihood (ML) analysis of the concatenated nucleotide sequences based on substitution rates using the General Time Reversible model

Maximum likelihood analysis of the sequences grouped the 41 isolates into several evolutionary clusters (Fig. 2). Among them, IIdA20G1 isolates from Heilongjiang formed one cluster separated from other isolates including IIdA20G1 isolates from Hebei. Another cluster was formed by IIdA15G1 isolates from Xinjiang. In contrast, there was no significant geographical clustering among IIdA19G1 isolates from Jiangsu, Shanghai and Guangdong (Fig. 2).

**Fig. 3:**
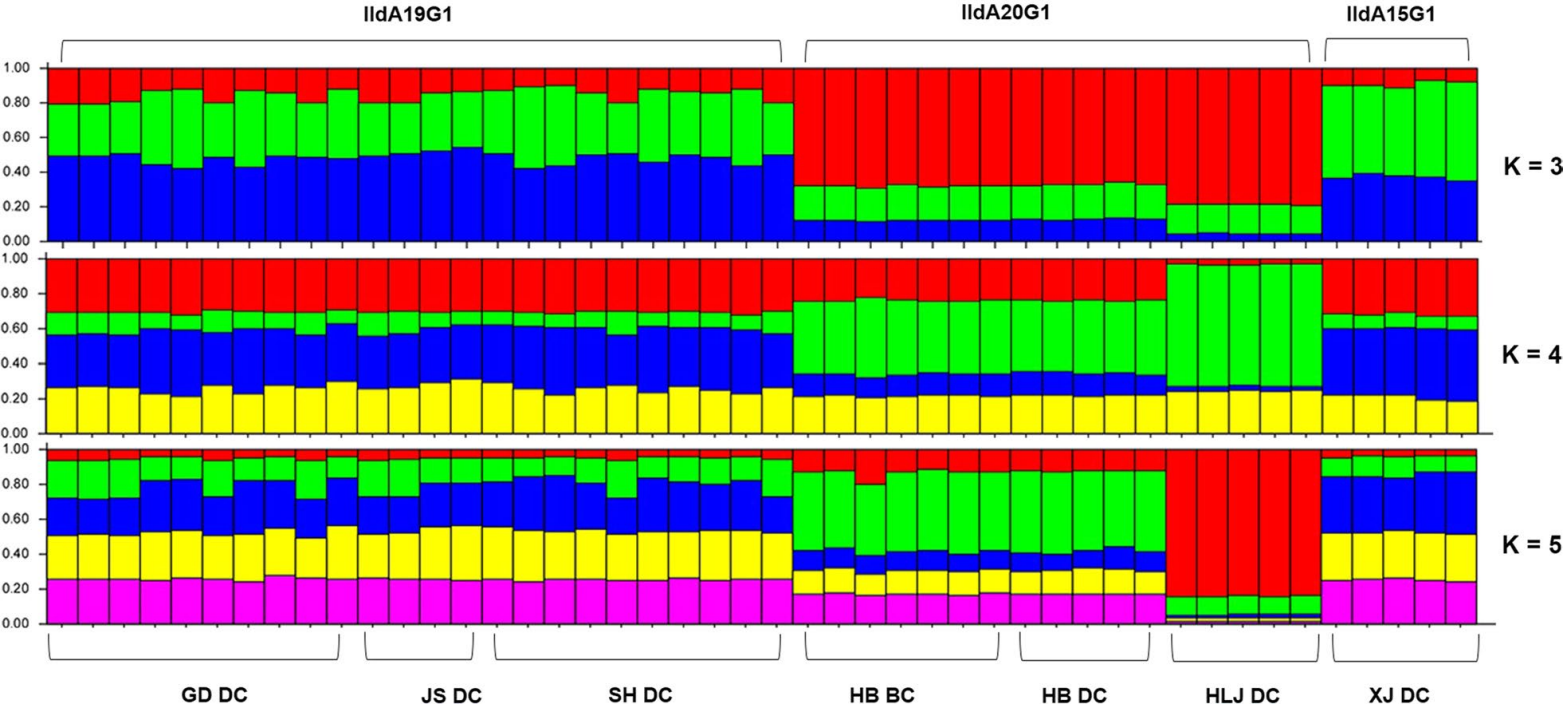
Substructure analysis of 46 *Cryptosporidium parvum* isolates inferred by Bayesian clustering of allelic data. K = 3, 4 and 5 were used in the analysis. *gp60* subtypes and geographical origins of the isolates are shown above and below the bars, respectively. *GD* Guangdong, *JS* Jiangsu, *SH* Shanghai, *HB* Hebei, *HLJ* Heilongjiang, *XJ* Xinjiang, *DC* dairy cattle, *BC* beef cattle

**Fig. 4:**
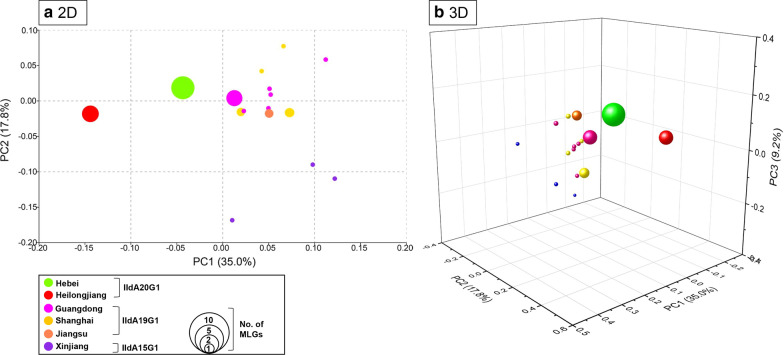
Results of the principal coordinate analysis of 41 *Cryptosporidium parvum* isolates based on pairwise distances (a 2D, b 3D). Each solid sphere represents an MLG. The color of the spheres indicates geographical origin of the isolates, while the size of the spheres represents the number of isolates

**Fig. 5:**
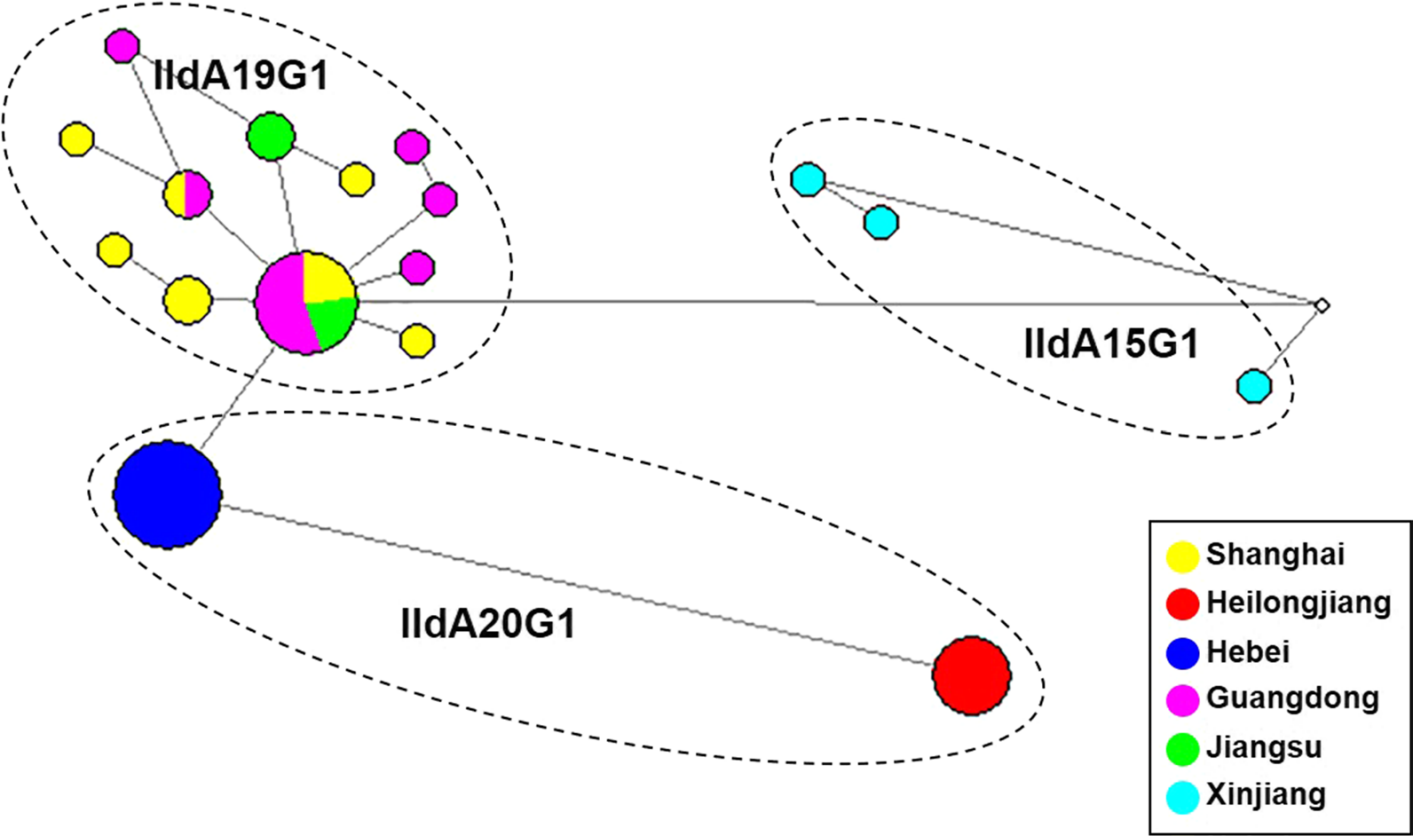
Phylogeny of 41 *Cryptosporidium parvum* isolates inferred by median-joining network analysis. The size of each circle is proportional to the number of isolates with the MLG, while the color of the circles indicates the geographical origin of the MLG

A similar result was obtained in STRUCTURE analysis of allelic data. At all K-values used in the analysis, the IIdA20G1 isolates from Heilongjiang were clearly separated from isolates of other regions, including those from Hebei that had the same *gp60* subtype. The best separation of subpopulations by *gp60* subtype was seen at a K-value of 3; all three *C. parvum* subtypes formed their own clusters (Fig. 3). In addition, regardless the K-values (3–5) used in the analyses, IIdA19G1 isolates from Guangdong, Shanghai and Jiangsu clustered together (Fig. 3). This was supported by the results of PCoA and median-joining network analyses, in which isolates from Heilongjiang, Hebei and Xinjiang formed their own clusters while those from Shanghai, Jiangsu and Guangdong clustered together (Figs. 4 and 5).

The results of *F*_st_ analysis supported the occurrence of geographically associated subpopulations of *C. parvum* IId subtypes. By *gp60* subtype, isolates of IIdA15G1, IIdA19G1 and IIdA20G1 were genetically segregated from each other with high statistical significance (Table 4). Within the IIdA20G1 subtype, there was a significant differentiation between isolates from Hebei and Heilongjiang (*χ*^2^ = 15.0, *df* =1, *P* < 0.0001). In contrast, the differentiation among IIdA19G1 isolates from Guangdong, Shanghai and Jiangsu was low. Compared with IIdA20G1 isolates from Heilongjiang, there was also reduced differentiation of between IIdA20G1 isolates from Hebei and IIdA19G1 isolates from Jiangsu and Shanghai (Table 5).

**Table 4:** Pairwise genetic distance (*F*_st_, below the diagonal) and *P*-values based on Chi-square test (above the diagonal) between subpopulations of *Cryptosporidium parvum* by *gp60* subtype

Subtype	IIdA19G1	IIdA20G1 (*χ*^2^, *df*, *P*)	IIdA15G1 (*χ*^2^, *df*, *P*)
IIdA19G1		(18.2, 12, < 0.0001)	(26.0, 14, < 0.0001)
IIdA20G1	0.14262		(18.0, 4, < 0.0001)
IIdA15G1	0.34028	0.32851

**Table 5:** Pairwise genetic distance (*F*_st_, below the diagonal) and *P*-values based on Chi-square test (above the diagonal) between subpopulations of *Cryptosporidium parvum* by geographical origin

Location	Guangdong	Jiangsu (*χ*^2^, *df*, *P*)	Shanghai (*χ*^2^, *df*, *P*)	Hebei (*χ*^2^, *df*, *P*)	Heilongjiang (*χ*^2^, *df*, *P*)	Xinjiang (*χ*^2^, *df*, *P*)
Guangdong		(8.3, 7, 0.00901 ± 0.0091)	(11.3, 10, 0.02703 ± 0.0194)	(6.7, 5, 0.23423 ± 0.0411)	(3.8, 5, < 0.0001)	(13.0, 8, < 0.0001)
Jiangsu	0.14401		(8.3, 7, 0.04505 ± 0.0244)	(5.8, 1, 0.01802 ± 0.0121)	(3.2, 1, < 0.0001)	(7.0, 4, < 0.0001)
Shanghai	0.05817	0.09422		(12.3, 6, 0.01802 ± 0.0121)	(7.8, 6, < 0.0001)	(12.0, 9, < 0.0001)
Hebei	0.02455	0.32678	0.08043		(15.0, 1, < 0.0001)	(13.0, 3, < 0.0001)
Heilongjiang	0.67935	0.85765	0.5247	0.87905		(8.0, 3, < 0.0001)
Xinjiang	0.36149	0.29526	0.28499	0.36733	0.47694	

## Discussion

The population genetic analysis of eight polymorphic loci has unravelled a high genetic diversity among isolates of *C. parvum* IId subtypes from different geographical areas in China. Although they were identical at the *gp60* locus, the IIdA19G1 isolates differed at most other genetic loci including *dz-hrgp*, *msc6-5*, *msc6-7*, *mucin1* and *rpgr*. Similarly, IIdA20G1 isolates from Hebei and Heilongjiang differed from each other at the *hsp70* locus, while IIdA15G1 isolates from Xinjiang differed from each other at the *dz-hrgp*, *hsp70*, *msc6-5* and *msc6-7* loci.

Results of the *LD* analysis indicate the presence of an epidemic genetic structure of *C. parvum* IId subtypes in the present study. This could be attributed to the high prevalence of *C. parvum* in calves as the result of concentrated animal feeding operations and limited number of IId subtypes in China [2]. Indeed, IIdA19G1 and IIdA15G1 are dominant subtypes in cattle in China [7, 32]. Previously, isolates of *C. parvum* IId subtypes from China, Egypt and Sweden were shown to have a clonal population structure with limited genetic recombination [25]. The discrepancy in the inference of population genetic structure between these two studies was largely due to whether the analysis has taken the over-representation of the same MLG in the study population into consideration. If this had taken into consideration, the previously reported clonal population of *C. parvum* IId subtypes could be in fact an epidemic population.

Significant geographical segregation was observed in the IIdA15G1 isolates from Xinjiang and the IIdA20G1 isolates from Heilongjiang based on phylogenetic, substructure, PCoA and *F*_st_ analyses. Previous reports indicated that most IIa isolates of *C. parvum* form country-specific populations. For example, an eBURST-based analysis revealed geographical differences among isolates from Uganda, Israel, Serbia, Turkey and New Zealand [33]. Similarly, a significant geographical segregation was also identified among 692 *C. parvum* isolates from Italy, Ireland and Scotland [13]. The same situation was also observed in IId isolates of *C. parvum* between China and Sweden in a previous MLST study [25]. Other studies, however, have failed to identify geographical segregation in *C. parvum* populations, but they were conducted over smaller geographical areas within a country [17, 18]. In the present study, diverse isolates were obtained from the southern, north-eastern and north-western regions of China, leading to the identification of unique subpopulations of *C. parvum* in the more geographically isolated Xinjiang and Harbin. In contrast, isolates from Shanghai, Jiangsu and Guangdong had frequent genetic exchanges with no significant geographical barriers among them. These regions were chosen with the consideration of both geographical representation and the intensity of cattle production. The three *C. parvum* subtypes examined in the study are the most common ones in China, responsible for over 90% *C. parvum* infections in cattle. Additional population genetic analysis of more *C. parvum* isolates from other areas and other subtypes is needed to support the observations in this study.

The presence of multiple MLGs on almost all farms in Guangdong, Shanghai and Jiangsu suggests the presence of a significant intra-farm genetic diversity in *C. parvum*. This was not revealed by *gp60*-based subtyping, as all isolates belonged to IIdA19G1. Nevertheless, this is in agreement with previous population genetic studies of *C. parvum* in European countries [12, 15, 19, 22, 25, 34]. This intra-farm genetic diversity of *C. parvum* in cattle may be attributed to frequent animal trade among farms, which is known to increase the heterogeneity of *C. parvum* and the complexity of infections [14, 19, 21].

## Conclusions

Despite the presence of only a limited number of *gp60* subtypes of *C. parvum* in cattle in China, a much higher genetic diversity is evident in MLST characterization of isolates at both farm and region levels. Nevertheless, biological selection has led to the dominance of limited numbers of geographically segregated MLGs of *C. parvum* in calves in China, with an apparent epidemic population structure. Currently, the veterinary and public health significance of this biological selection of *C. parvum* subpopulations is not entirely clear. Efforts should be made to monitor the genetic evolution of this unique zoonotic pathogen in China.

## Supplementary information

**Additional file 1: Table S1.** *Cryptosporidium parvum* isolates used in this study and their genetic identity at eight loci.

## Data Availability

The data supporting the conclusions of this article are included within the article. Representative nucleotide sequences generated from this study were deposited in the GenBank database under the accession numbers MT303107-MT303131.

## Abbreviations

PCR: Polymerase chain reaction
*gp60*: 60-kDa glycoprotein
*msc6-7*: Serine repeat antigen
*rpgr*: Retinitis pigmentosa GTPase regulator
*msc6-5*: Hypothetical trans-membrane protein
*dz-hrgp*: Hydroxyproline-rich glycoprotein
*chom3t*: T-rich gene fragment
*hsp70*: 70-kDa heat-shock protein
*mucin1*: Mucin-like protein
LD: Linkage disequilibrium
LE: Linkage equilibrium
MLG: Multi-locus genotype
*Rms*: Recombination events
