# Supplementary material for: Population structure and geographical segregation of Cryptosporidium parvum IId subtypes in cattle in China
Source: Parasit Vectors. 2020 Aug 18;13:425. doi: 10.1186/s13071-020-04303-y (PMC7437029; doi:10.1186/s13071-020-04303-y)
Supplement: Supplementary file 1 — Additional file 1: Table S1. Cryptosporidium parvum isolates used in this study and their genetic identity at eight loci. [file 13071_2020_4303_MOESM1_ESM.doc]

**Additional file 1: Table S1.** *Cryptosporidium parvum* isolates used in this study and their genetic identity at eight loci

| **Sample ID** | **Location** | **Host** | ***gp60*** | ***chom3t*** | ***dz-hrgp*** | ***hsp70*** | ***msc6-5*** | ***msc6-7*** | ***mucin1*** | ***rpgr*** | **MLGs** |
| --- | --- | --- | --- | --- | --- | --- | --- | --- | --- | --- | --- |
| 22597 | Guangdong (Farm D) | Dairy cattle | 2 | 1 | 2 | 1 | 1 | 1 | 1 | 1 | MLG3 |
| 22598 |  |  | 2 | 1 | 2 | 1 | 1 | 1 | 1 | 1 | MLG3 |
| 22971 |  |  | 2 | 1 | 2 | 1 | 1 | 1 | 1 | 1 | MLG3 |
| 22972 |  |  | 2 | 1 | 2 | 1 | 1 | 1 | 1 | 3 | MLG10 |
| 22973 |  |  | 2 | 1 | 2 | 1 | 1 | 1 | 1 | 4 | MLG11 |
| 6294 | Guangdong (Farm E) |  | 2 | 1 | 2 | 1 | 1 | 1 | 1 | 1 | MLG3 |
| 6307 |  |  | 2 | 1 | 4 | 1 | 1 | 1 | 1 | 1 | MLG4 |
| 6290 |  |  | 2 | 1 | 2 | 1 | 1 | 1 | 3 | 1 | MLG7 |
| 6295 |  |  | 2 | 1 | 2 | 1 | 1 | 1 | 1 | 1 | MLG3 |
| 6316 |  |  | 2 | 1 | 2 | 1 | 1 | 2 | 3 | 1 | MLG9 |
| 12531 | Hebei  (Farm H) | Beef cattle | 1 | 1 | 2 | 1 | 1 | 1 | 1 | 1 | MLG2 |
| 12534 |  |  | 1 | 1 | 2 | 1 | 1 | 1 | 1 | 1 | MLG2 |
| 12536 |  |  | 1 | 1 | – | 1 | 1 | 1 | 1 | 1 | – |
| 12539 |  |  | 1 | 1 | 2 | 1 | 1 | 1 | 1 | 1 | MLG2 |
| 12540 |  |  | 1 | 1 | 2 | 1 | 1 | 1 | 1 | 1 | MLG2 |
| 24694 |  |  | 1 | 1 | 2 | 1 | 1 | 1 | 1 | 1 | MLG2 |
| 24696 |  |  | 1 | 1 | 2 | 1 | 1 | 1 | 1 | 1 | MLG2 |
| 24712 | Hebei  (Farm G) | Dairy cattle | 1 | 1 | 2 | 1 | 1 | 1 | 1 | 1 | MLG2 |
| 24716 |  |  | 1 | 1 | 2 | 1 | 1 | 1 | 1 | 1 | MLG2 |
| 24720 |  |  | 1 | 1 | 2 | 1 | 1 | 1 | 1 | 1 | MLG2 |
| 24721 |  |  | 1 | 1 | 2 | 1 | 1 | 1 | – | 1 | – |
| 24725 |  |  | 1 | 1 | 2 | 1 | 1 | 1 | 1 | 1 | MLG2 |
| 11692 | Heilongjiang (Farm F) | Dairy cattle | 1 | 1 | 1 | 2 | 1 | 1 | 1 | 1 | MLG1 |
| 11698 |  |  | 1 | 1 | 1 | 2 | 1 | 1 | 1 | 1 | MLG1 |
| 11717 |  |  | 1 | 1 | 1 | 2 | 1 | 1 | 1 | 1 | MLG1 |
| 11720 |  |  | 1 | 1 | 1 | 2 | 1 | 1 | 1 | 1 | MLG1 |
| 11722 |  |  | 1 | 1 | 1 | 2 | 1 | 1 | 1 | 1 | MLG1 |
| 3702 | Jiangsu  (Farm A) | Dairy cattle | 2 | 1 | 2 | 1 | 1 | 1 | 1 | 1 | MLG3 |
| 4119 |  |  | 2 | 1 | 2 | 1 | 1 | 1 | 1 | 1 | MLG3 |
| 17357 |  |  | 2 | 1 | 2 | 1 | 1 | 2 | 1 | 1 | MLG5 |
| 17365 |  |  | 2 | 1 | 2 | 1 | 1 | 2 | 1 | 1 | MLG5 |
| 18252 | Shanghai  (Farm B) | Dairy cattle | 2 | – | 2 | 1 | 1 | 2 | – | – | – |
| 18421 |  |  | 2 | 1 | 2 | 1 | 1 | 2 | 1 | 2 | MLG6 |
| 18233 |  |  | 2 | 1 | 5 | 1 | 2 | 1 | 1 | 1 | MLG13 |
| 18242 |  |  | 2 | 1 | 2 | 1 | 2 | 1 | 1 | 1 | MLG14 |
| 18250 |  |  | 2 | 1 | 2 | 1 | 1 | 1 | 1 | 1 | MLG3 |
| 18408 | Shanghai  (Farm C) |  | 2 | 1 | 2 | 1 | 1 | 1 | 2 | 1 | MLG8 |
| 18409 |  |  | 2 | 1 | 2 | 1 | 1 | 1 | 3 | 1 | MLG7 |
| 18410 |  |  | 2 | 1 | 2 | 1 | 2 | 1 | 1 | 1 | MLG14 |
| 18426 |  |  | 2 | 1 | 2 | 1 | 1 | 1 | 4 | 1 | MLG12 |
| 17344 |  |  | 2 | 1 | 2 | 1 | 1 | 1 | 1 | 1 | MLG3 |
| 33683 | Xinjiang  (Farm I) | Dairy cattle | 3 | 1 | – | – | 1 | 3 | 1 | – | – |
| 33685 |  |  | 3 | 1 | 2 | 1 | 1 | 3 | 1 | 1 | MLG16 |
| 33686 |  |  | 3 | – | 2 | – | 1 | 3 | 1 | 1 | – |
| 33687 |  |  | 3 | 1 | 3 | 1 | 1 | 3 | 1 | 1 | MLG17 |
| 33688 |  |  | 3 | 1 | 2 | 3 | 3 | 1 | 1 | 1 | MLG15 |
